# Characterization and In Silico Analysis of The Structural Features of G-CSF Derived from Lysates of *Escherichia coli*

**DOI:** 10.22074/cellj.2020.6158

**Published:** 2019-07-31

**Authors:** Sharareh Peymanfar, Rasoul Roghanian, Kamran Ghaedi, Sayed-Hamid Zarkesh-Esfahani, Reza Yari

**Affiliations:** 1 Department of Biology, Faculty of Science, University of Isfahan, Isfahan, Iran; 2 Department of Biology, Borujerd Branch, Islamic Azad University, Borujerd, Iran

**Keywords:** Characterization, *E. coli*, Granulocyte Colony-Stimulating Factor

## Abstract

**Objective:**

Granulocyte colony-stimulating factor (G-CSF) has a wide variety of functions including stimulation of hematopoiesis and proliferation of granulocyte progenitor cells. Recombinant human G-CSF (rh-G-CSF) is used for treatment of neutropenia in patients receiving chemotherapy. The mature bloodstream neutrophils express G-CSF receptor (G-CSFR), presenting a significant and specific mechanism for circulating G-CSF clearance. Computational studies are essential bioinformatics methods used for characterization of proteins with regard to their physicochemical properties and 3D configuration, as well as protein–ligand interactions for recombinant drugs. We formerly produced rh-G-CSF in *E. coli* and showed that the isolated protein had unacceptable biological activity in mice. In the present paper, we aimed to characterize the purified rh-G-CSF by analytical tests and developed an *in vivo* model by computational modelling of G-CSF.

**Materials and Methods:**

In this experimental study, we analyzed the purified G-CSF using the analytical experiments. Then, the crystalline structure was extracted from Protein Data Bank (PDB) and molecular dynamics (MD) simulation was performed using Gromacs 5.1 package under an Amber force field. The importance of amino acid contents of G-CSF, to bind the respective receptor was also detected; moreover, the effect of dithiothreitol (DTT) used in G-CSF purification was studied.

**Results:**

The results revealed that characteristics of the produced recombinant G-CSF were comparable with those of the standard G-CSF and the recombinant G-CSF with the residual amino acid was stable. Also, purification conditions (DTT and existence of extra cysteine) had a significant effect on the stability and functionality of the produced G-CSF.

**Conclusion:**

Experimental and in silico analyses provided good information regarding the function and characteristics of our recombinant G-CSF which could be useful for industrial researches.

## Introduction

The granulocyte colony stimulating factor (G-CSF) serves a directing role in the growth, differentiation, and motivation of neutrophils and their precursors in the immune system (1). Likewise, G-CSF has been effectively utilized in patients undergoing intensive chemotherapy; further, it might be utilized to recover the immune system in the patients with HIV, pneumonia, and febrile neutropenia (2-4). The recombinant human G-CSF (rh-GCSF) was expressed in engineered *E. coli* and affirmed in chemotherapy-induced neutropenia by the U.S Food and Drug Administration (FDA) in 1991 for clinical usage (5). 

Previous studies described various protocols for assessment of rh-G-CSF. These protocols used different chromatography columns and diverse detergents for purification of rh-G-CSF (6-8). Nowadays, purification of proteins fused to intein tags is an easy task. N-terminus or C-terminus of the target protein binds the intein tags. To achieve this purpose and also to double the expression of target protein, in our previous study, we linked 2 copies of *G-CSF* gene with 2 different intein sequences and attained target protein in two forms: G-CSF and G-CSF plus cysteine (9, 10). 

Furthermore, since intein tags may interfere with G-CSF binding to its receptor and therefore, disrupt its biological activity, it is necessary to simulate molecular dynamics (MD) to check the presence of these tags and examine their effect on G-CSF binding to G-CSFR. The receptor of hematopoietic cytokine G-CSF is a member of a family of proteins referred to as the "family of cytokine receptors" and characterized by the existence of a 200-residue ligand-binding module. Alternatively, it is required to assess the effect of purification process on the protein stability and its receptor binding. To answer this question, computational studies were used in the present research. It should be mentioned that such study has not been conducted on G-CSF protein so far. The use of computational tools for determination of a new protein structure represents the most actual alternative to the experimental procedures. As relates to characterization of physicochemical and structural properties of a protein, there is no doubt that in silico methods can resolve complications made by purification techniques (11-15). 

Considering very different properties of commercially available rh-G-CSF proteins, we constructed a model of the isolated G-CSF and its G-CSFR. Previously, we produced G-CSF with intein tags and purified it using DTT and pH exchange. So, the present work was designed to identify functional and structural changes, especially with respect to the extra amino acid residue of G-CSF, and intein tags as well as the presence of DTT in protein solution, and finally compare the results obtained for natural and purified G-CSF. We studied G-CSF characteristics, created our models with respect to the improved dynamic modelling of rh-G-CSF and studied the effect of DTT and the residual cysteine on G-CSF stability and function. 

## Materials and Methods

### Designing the DNA fragment encompassing rh-GCSF coding sequence, intein, PelB, and intervening sequences of Trp operon 

**Fig.1:**
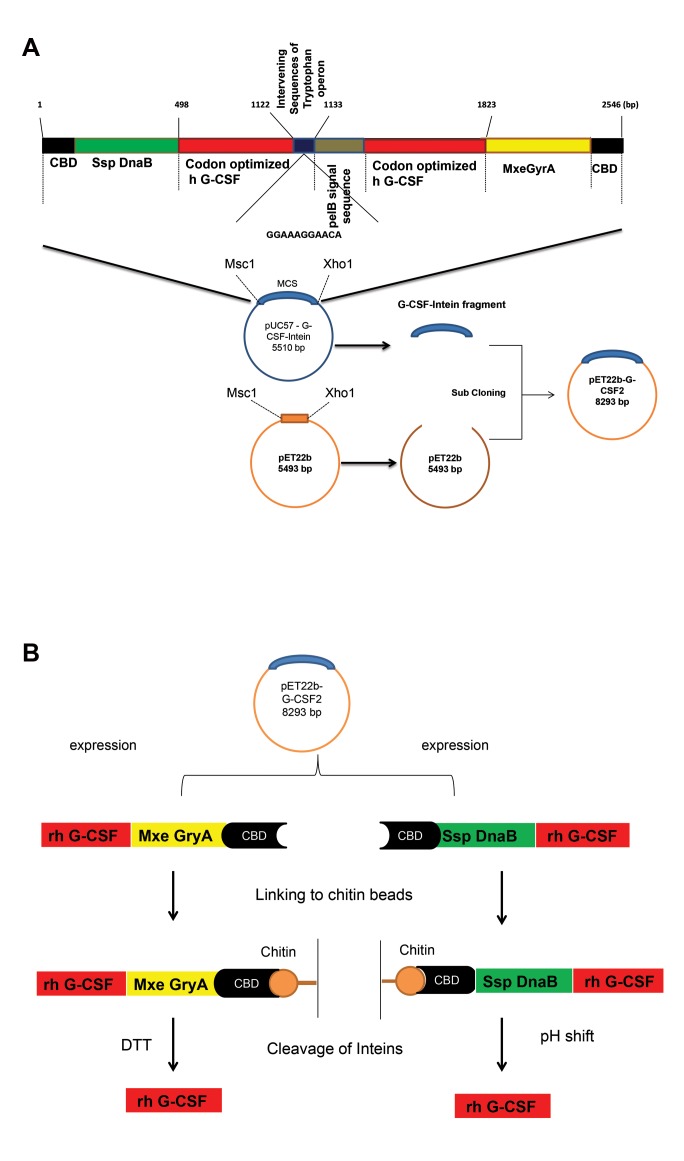
Overall schema of 2800 bp expression vector with *g-csf* gene and 2 different inteins genes, pelB signal sequence and intervening sequence. **A.** Schematic representation of the vector construction and the expression of the N-terminal fusion and C-terminal fusion proteins and **B.** In C-terminal fusion protein, intein2-CBD was fused to the C-terminus of h G-CSF, allowing the cleavage of h G-CSF from G-CSF-intein2 using DTT. In N-terminal fusion protein, CBD-intein1 was fused to the N-terminus of h G-CSF, allowing the cleavage of h G-CSF from intein1- G-CSF through a pH and temperature shift. G-CSF; Granulocyte colony-stimulating factor.

Two tandems of G-CSF sequences linked to inteins (CBD-Ssp DnaB and Mxe GryA-CBD) were described in a previous study. This fragment purchased from GENESCRIPT Company (USA) was acquired in the pUC57 vector located at *5'MscI* and 3' *XhoI* (14). In the next step, this fragment was inserted into the pET22b vector at the same sites in the downstream of the pelB signal sequence (Fig .1A) (9). 

### Rh-G-CSF production and purification

The cloning, expression and intein-mediated purification were performed in pET-22b (+) vector (9, 10, 16). Purification was performed according to the template shown in Figure 1B (9).

### Detection of *E. coli* host cell proteins

*E. coli* host cell proteins (HCP) were studied by an immunoenzymetric assay kit (Cygnus, USA) and an ELISA reader (Amersham, USA). According to the manufacturer’s protocol, 25 µL of each sample was loaded into each well. Next, 100 µL of *E. coli* antibody conjugated to horseradish peroxidase (HRP, Abcam, UK) was pipetted into each well. Then, these wells were covered and incubated on a rotator at 400-600 rpm for 90 minutes at room temperature (24 ± 4°C). The content of each well was dumped into waste. The wells were blotted softly but firmly tapped over an absorbent paper to remove most of the residual liquid. The wells were filled generously to overflow with the diluted wash solution using a squirt bottle or pipetting in 350 µL. Washing was repeated. From the bottom outside of the microtiter wells, any fluid residue that could interfere in the reading step, was wiped off. Then, 100 µL of the 3,3’,5,5’-tetramethylbenzidine (TMB) substrate was pipetted and incubation was performed at room temperature for 30 minutes. Finally, 100 µL of the stop solution was pipetted. Absorbance was read at 450/650 nm. The microplate reader drew one standard curve and automatically calculated the amount of HCP in the purified protein (17). 

### Measurement of bacterial endotoxin contamination 

The amount of bacterial endotoxin was determined by the LAL kit (Lonza, Switzerland) and an ELISA reader (Amersham, USA). Carefully, 50 µL of the purified protein was dispensed into suitable tubes that were endotoxin-free. These tubes were kept in a 37°C water bath. Blank tubes plus four endotoxin standards were run in duplicate. The blank tubes contained 50 µL of the LAL reagent water instead of the sample. At the same time, to the reaction vessel, 50 µL of LAL was added. Then, 100 µL of the substrate solution was added to the tubes (prewarmed to 37 ± 1°C) after 10 minutes. After 16 minutes, 100 µL of the stop reagent was added and mixed well. Absorbance of reaction in each tube was read at 405/410 nm by the ELISA reader using distilled water to adjust the photometer to the zero absorbance. The ELISA reader drew one standard curve and automatically calculated the amount of endotoxin in the purified protein (17).

### Impurities with charges that were different from those of Filgrastim (Isoelectric focusing)

Isoelectric focusing (IEF) was performed using a pH 3-10 IEF gel (Invitrogen, Grand Island, NY) (17, 18). The electrophoresis system (Multiphor II, Amersham, USA) was fitted out with a buffer tank, a cooling plate, an electrode holder, and electrophoresis (EPH)/IEF electrodes. Experiments were performed using 7-cm immobilized pH gradient (IPG) stripes (pH=3-10). The system worked at room temperature. The pH gradient was 4.5-8.0 and an isoelectric point (pI) calibration solution was prepared in the pI range of 2.5-6.5. The standard and the sample were put on the stripes. In this test, proteins were moving in the gel stripes and finally, the purified protein and standard protein were stopped at their pI. If there was any impurity in the purified protein, it would move in the gel and could be stopped at its pI. The Filgrastim standard was obtained from Pooyesh Darou Company (Iran). 

### Peptide mapping with RP-HPLC 

Peptide map analysis is an important analytical technique widely used to verify protein primary structures. The method is capable of specifically detecting and quantifying structural alterations in the recombinant proteins, such as those derived from N-terminal blockage, oxidation, proteolysis, or amino acid substitutions. To identify the structure and fundamental variations, RP-HPLC was used (17, 18). In this test, standard and sample proteins were digested by glutamyl endopeptidase for 18 hours at 37°C and then analyzed by HPLC. The HPLC system (Agilent, USA) included a system controller, a pump, a degasser, the auto-sampler, and a photodiode array (PDA) detector. The detector was set at 214 nm and the peak regions were integrated by computational analyses. Experiments were done using a C18 column (100× 2.1 mm, 5 µm particle size, with 300 A° pore size). The HPLC test was performed at 60°C, using acetonitrile as the mobile phase. The standard and the sample were at the concentration of 25 µg per 10 µL, and all determinations were carried out in triplicate. The flow rate was 0.2 mL/minutes. Retrieval of protein sequences and 3D structures The protein sequences of G-CSF and G-CSFR were achieved from UniProt (http://www.uniprot.org/). The protein sequences were retrieved in the FASTA format in order to be analyzed by the computational methods. Also, the crystal structure of G-CSF and G-CSFR from the PDB data storage site with the codes 1GNC and 2D9Q, was obtained; finally, it was used as the primary structure for the MD simulation. Likewise, sequences of inteins (Ssp DnaB and Mxe GryA) were obtained from InBase database (New England Biolabs Intein Database available at http://www.inteins.com/) and used in the MD Simulation (19-21).

### Molecular dynamics simulation 

MD simulation was performed by Gromacs 4.6.4 package using GROMOS96 (53a6) (19). In this test, effect of DTT (40 mM) and pH exchange was studied on the protein structure and stability and also the effect of extra cysteine on G-CSF stability and its GCSF-R binding was evaluated. The protein topology file for Gromacs was provided utilizing the program Topolbuild, developed by Bruce D. Ray. All systems were solvated by explicit solvents (TIP3P) and counter ions were added to neutralize each system. Next, the energy minimization (EM) was performed by the steepest descent algorithm at tolerance value of 100 kJ/mol.nm. Also, EM was followed by the equilibration with position restraint on the protein molecule for 0.5 ns using Constant temperature and volume (NVT) and Constant temperature and pressure (NPT) ensembles by standard coupling methods. Then, main runs were performed without any restraint on the protein molecule, trajectories were generated with a time interval of 0.01 ns, and frames were saved at every 0.01 ns. Particle mesh ewald (PME) summation was used to assess long-range Coulomb interactions (20). In the equilibration and subsequent production runs, the internal degrees of freedom of the solvent molecules were restrained by the SHAKE algorithm (21), and all bond lengths were restrained in the macromolecules via the LINCS algorithm (22). All analyses were provided by Gromacs toolbox and the images were produced by PyMol (23) and LigPlot (24). 

## Results

### Detection of *E. coli* host cell proteins in the purified rh-G-CSF solution 

*E. coli* HCP were detected by the immunoenzymetric assay kit (Cygnus, USA). The results indicated that HCP was less than 100 ppm/dose and thus, they were in the permissible range (data not shown). 

### Bacterial endotoxins

The number of bacterial endotoxins was determined by the LAL kit (Lonza, Switzerland). The results showed that the amount of *E. coli* endotoxins was less than 2 IU/mg protein. 

### Impurities with charges that were different from those of Filgrastim (Isoelectric focusing) 

**Fig.2:**
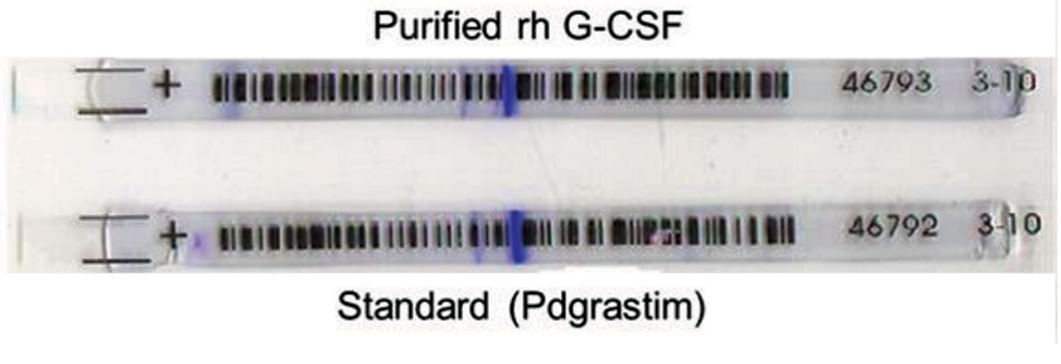
Isoelectric focusing of purified rh G-CSF and PDgrastim (standard) in the strips of pH=3-10. Both of the standard and purified proteins were stopped in pH=6.1 and no impurity or no band was found. G-CSF; Granulocyte colony-stimulating factor.

IEF was performed using a pH=3-10 IEF gel. Filgrastim main bond in the standard and purified sample was stopped at pH=6.1; also, no band was more intense than the chief one in the electropherogram attained with the reference solution (PDgrastim) (Fig .2). 

### Peptide mapping 

**Fig.3:**
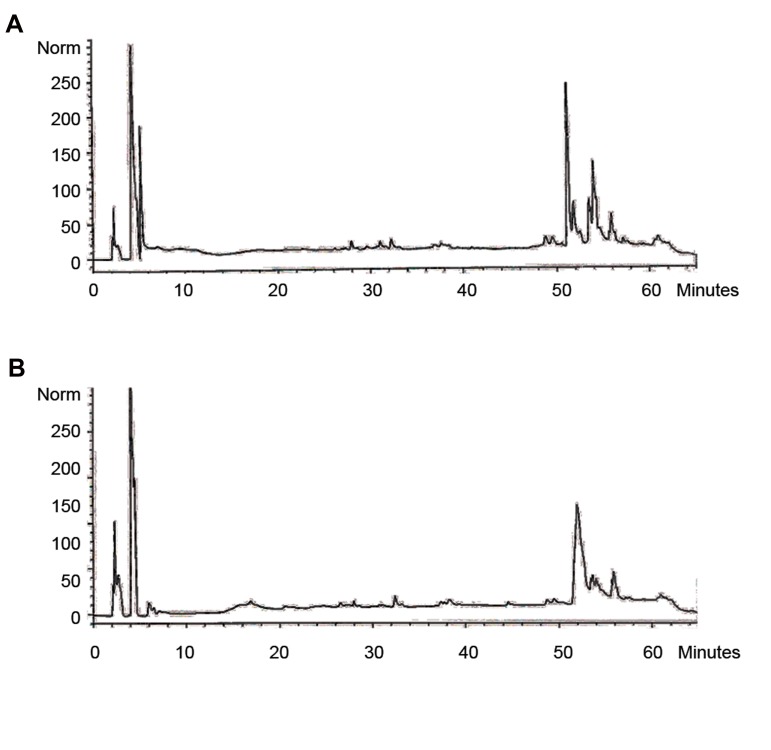
The reversed phase HPLC chromatogram for the Endopeptidase digest of purified rh G-CSF in comparison with standard rh G-CSF. **A.** Peptide map chromatograms of PDgrastim and **B.** Purified rh G-CSF. G-CSF; Granulocyte colony-stimulating factor.

To confirm the protein structure and identify modifications, peptide mapping and RP-HPLC were done (18). The chromatogram attained with the reference solution was similar to that of the purified G-CSF. The chromatogram obtained with the test solution corresponded to that of the chromatogram achieved with the reference, but they were not exactly the same (Fig .3). This discrepancy could be due to the fact that the enzyme did not affect the purified protein or changed the amino acid content. 

### Molecular dynamic simulation 

The crystal structure of proteins was attained from PDB. Visualization of the proteins model was done by Chimera (version 1.8). To determine the structural and functional differences between G-CSF and cysteine- G-CSF and to find their differences in binding GCSF-R, MD simulation studies were performed. The simulations were carried out for all of the mentioned structures, as mentioned in the Materials and Methods section, under explicit solvent conditions. The trajectory results obtained from MD simulation were evaluated for RMSD and RMSF. 

### The effect of dithiothreitol on the protein stability and receptor binding

**Fig.4:**
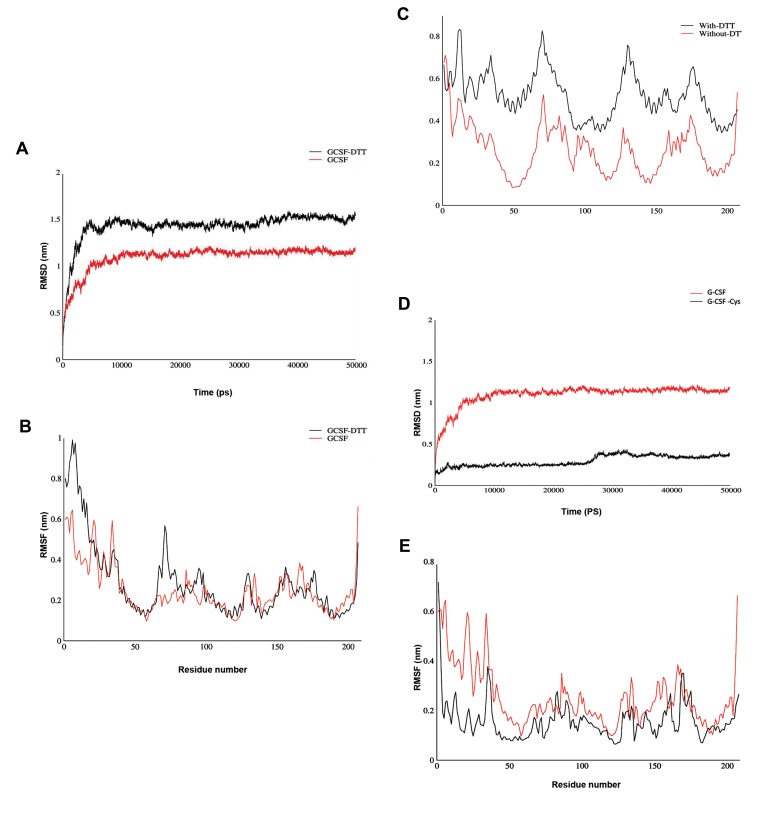
Results of molecular dynamic simulation of RMSF and RMSD diagram. **A.** Comparison of RMSD changes. The simulation of G-CSF protein in the presence and absence of DTT, **B.** Comparison of the RMSF diagram of G-CSF in the presence and absence of DTT, **C.** RMSF diagram of GCSF protein in complex with GCSFR in the presence of DTT, **D.** The RMSD variations associated with the natural GCSF protein and GCSF have Cysteine amino acid, and **E.** RMSF diagram shows flexible amount of single amino acids for normal and atypical protein (contains cysteine). RMSF; Root-mean-square fluctuation, RMSD; Root-mean-square deviation, G-CSF; Granulocyte colony stimulating factor, and DTT; Dithiothreitol.

The Figure 4A depicts the RMSD variations during the simulation of G-CSF protein in the presence and absence of DTT. As shown in Figure 4A, protein in the presence of DTT was more unstable and its RMSD was greater than that in the non-DTT state. Likewise, the RMSF graph showed that in the presence of DTT, the protein’s flexibility was increased in certain regions (Fig .4B).

As shown in the RMSF diagram, in the residues 1 to 40, as well as 65 to 80, the presence of DTT led to a dramatic increase. As shown by the RMSD and RMSF diagrams, the presence of DTT increased protein flexibility. Additionally, the stability of the G-CSF protein, when bound to GCSF-R, was monitored in the presence of DTT. For this reason, in this part, the RMSF diagram of the GCSF protein was analyzed in the complex mode with GCSFR in the presence of DTT (Fig .4C).

As shown in this diagram, DTT increased the protein’s flexibility in all its amino acids. This increase in flexibility could probably cause the GCSF- GCSFR complex to become unstable and reduce the binding affinity between the two proteins. 

### The effect of the residual cysteine on the protein stability and receptor binding 

After G-CSF purification, it was possible to keep the extra cysteine in the N-terminal of G-CSF. Hence, to study the effect of this amino acid on the protein stability and receptor binding, we performed the computational analysis. The Figure 4D shows the variations of the RMSD associated with the natural GCSF protein and the cysteine residue. As shown, the presence of cysteine residue in the protein significantly reduced the RMSD level and stabilized the protein over time. 

The graph shows the flexibility of the residues for a natural and atypical protein (containing cysteine). As shown in the RMSF diagram, the total flexibility of the structure in the atypical state was reduced, as compared with the normal one, and this reduction was significant in the early amino acid sequence of the protein. Analysis of the two above-mentioned graphs shows that the presence of cysteine residues reduced the flexibility and stability of the G-CSF structure (Fig .4E). 

## Discussion

rh-G-CSF is widely used as a useful hematopoietic growth factor. Therefore, production of rh-G-CSF seems to be required. Hence, it was decided to analyze rh-G-CSF that had already been produced in *E. coli* with laboratory and in silico methods. Formerly, we produced G-CSF linked to intein (9). The intein segments were preferably used for better expression and purification due to their low molecular weight, resulting in a high percentage of small G-CSF as fusion protein. Xie et al. (10) cloned OG2 antimicrobial peptide in the pTWIN1 vector with two different intein segments and achieved high-rate expression and production of the recombinant protein. They achieved purified proteins with extra cysteine in the N-terminal. We also implemented this procedure to purify rh-G-CSF with chitin beads, achieving G-CSF and cysteine-GCSF. Then, we analyzed the purified G-CSF for impurities using IEF and HCP determination tests, also the structure was analyzed using peptide mapping. Our results were comparable with those achieved by Kim et al. (18). This was because they could produce G-CSF in *E. coli* with good quality and no impurity. Additionally, we investigated the effect of DTT (used for purification) and extra cysteine on stability and function of G-CSF. The results displayed that the purified G-CSF had characteristics similar to those of the standard, and DTT reduced G-CSF stability and increased its flexibility. Also, our studies showed that the residual cysteine increased the stability and reduced flexibility; therefor e , it might not affect receptor binding. The results of computational studies of this protein were shown to be consistent with those of laboratory studi e s and the biological activity of this protein. In this way, the presence or absence of cysteine could not have any important effect on the biological activity of protein. Comparison of GCS-F and cysteine-GCSF results revealed that both of them were stable and probably had similar functionality. It was even imaginable that the modified protein had higher biological activity; w e previously o bserved that cystein e -GCSF biological activity was higher than that of the standard G-CSF (9). 

## Conclusion

The inform ation displayed here may be important to biophar maceutical companies to produce new recombinan t proteins using new techniques. Despite various ap plications of MD simulation in protein engineering, no similar research was done on this protein. Therefore, studying the structural dynamic properties of this prote in under different conditions provided a new opportunity to study the stability and function of G-CSF.
